# Preclinical Therapy with Vitamin D3 in Experimental Encephalomyelitis: Efficacy and Comparison with Paricalcitol

**DOI:** 10.3390/ijms22041914

**Published:** 2021-02-15

**Authors:** Luiza Ayumi Nishiyama Mimura, Thais Fernanda de Campos Fraga-Silva, Larissa Ragozzo Cardoso de Oliveira, Larissa Lumi Watanabe Ishikawa, Patrícia Aparecida Borim, Carla de Moraes Machado, José de Anchieta de Castro e Horta Júnior, Denise Morais da Fonseca, Alexandrina Sartori

**Affiliations:** 1 Department of Chemical and Biological Sciences, Institute of Biosciences, São Paulo State University (UNESP), Botucatu 18618-689, Brazil; thaisfragasilva@gmail.com (T.F.d.C.F.-S.); larissa.ragozo@unesp.br (L.R.C.d.O.); larissalumi@gmail.com (L.L.W.I.); alexandrina.sartori@unesp.br (A.S.); 2 Botucatu Medical School, Department of Tropical Diseases and Image Diagnosis, São Paulo State University (UNESP), Botucatu 18618-687, Brazil; patricia.borim@hotmail.com; 3 Department of Structural and Functional Biology, Institute of Biosciences, São Paulo State University (UNESP), Botucatu 18618-689, Brazil; moraescm90@gmail.com (C.d.M.M.); anchieta.e@unesp.br (J.d.A.d.C.eH.J.); 4 Department of Immunology, Institute of Biomedical Sciences, University of Sao Paulo (USP), São Paulo 05508-000, Brazil; denisefonseca@usp.br

**Keywords:** experimental autoimmune encephalomyelitis, vitamin D3, vitamin D analog, paricalcitol, inflammation, dendritic cells, gut

## Abstract

Multiple sclerosis (MS) is a chronic demyelinating disease of the central nervous system (CNS). MS and its animal model called experimental autoimmune encephalomyelitis (EAE) immunopathogenesis involve a plethora of immune cells whose activation releases a variety of proinflammatory mediators and free radicals. Vitamin D3 (VitD) is endowed with immunomodulatory and antioxidant properties that we demonstrated to control EAE development. However, this protective effect triggered hypercalcemia. As such, we compared the therapeutic potential of VitD and paricalcitol (Pari), which is a non-hypercalcemic vitamin D analog, to control EAE. From the seventh day on after EAE induction, mice were injected with VitD or Pari every other day. VitD, but not Pari, displayed downmodulatory ability being able to reduce the recruitment of inflammatory cells, the mRNA expression of inflammatory parameters, and demyelination at the CNS. Lower production of proinflammatory cytokines by lymph node-derived cells and IL-17 by gut explants, and reduced intestinal inflammation were detected in the EAE/VitD group compared to the EAE untreated or Pari groups. Dendritic cells (DCs) differentiated in the presence of VitD developed a more tolerogenic phenotype than in the presence of Pari. These findings suggest that VitD, but not Pari, has the potential to be used as a preventive therapy to control MS severity.

## 1. Introduction

Multiple sclerosis (MS) has been described as an inflammatory autoimmune disease that compromises the central nervous system (CNS) integrity and functioning. It usually begins between the ages of 20 and 40 years old, with a higher incidence among women and African Americans. It is considered to be the dominant cause of non-traumatic disability in young adults and affects approximately 2.5 million people worldwide, causing substantial socioeconomic impact [1,2]. Although the etiology of MS remains unknown, it is probably multifactorial involving mostly genetic and environmental factors. The notion that MS might be triggered by an infection is not new and this possibility derives from the association of viruses with post-infectious encephalitis [3], and the presence of high concentrations of IgG in the brain and cerebrospinal fluid of patients [4,5]. This view is reinforced by the association of several viral infections with demyelination in humans and the fact that certain virus infections also trigger demyelinating lesions in experimental animals [6]. The finding that MS is more prevalent in areas farther away from the equator, and therefore less sunny [7], raises an important question concerning the relevance of sun exposure to MS development. The most prevalent concept is that ultraviolet (UV) light exposition allows enough vitamin D3 (VitD) biosynthesis which is an essential internal immunomodulator [8]. Numerous studies have confirmed low VitD levels in MS patients and its correlation with disease severity and a higher rate of recurrences [9]. Lower VitD levels are also present in other autoimmune pathologies [10]. These findings are consistent with the remarkable systemic immunomodulatory potential [11] and neuroprotective ability of VitD [12,13], suggesting possible benefits for MS patients [14,15,16]. However, despite the considerable interest in using VitD as an add-on therapy for MS, and the numerous clinical assays previously performed, the benefits of its supplementation in patients are still a matter of debate and deserve further investigation [17,18]. Much of what is known about pathogenesis and alternative therapeutics for MS came from its corresponding animal model known as experimental autoimmune encephalomyelitis (EAE). This preclinical model is usually induced in rodents by immunization with CNS antigens in the presence of complete Freund’s adjuvant (CFA) and pertussis toxin [19,20]. Data from human and experimental MS have classically suggested that this pathology is initiated by activation of myelin-reactive Th1, Th17, and cytotoxic T lymphocytes in peripheral lymphoid organs [21,22]. More recently, a great deal of attention has been focused on the antibody-independent contribution of B cells [23] and the involvement of Th17 cells in MS evolution [24]. The contribution of Th17 cells takes place in different disease stages including during blood–brain barrier (BBB) permeabilization [25,26].

The fundamental concepts sustaining the indication of VitD for MS therapy were established by using the EAE model. Lamire and Archer (1991) [27], by using SJL female mice immunized with a spinal cord homogenate, demonstrated that the administration of VitD every other day for 15 days starting three days before disease induction significantly prevented EAE development. Cantorna et al. (1996) [28] showed that VitD could be both, prophylactic and therapeutic in B10.PL mice immunized with myelin basic protein. These authors also suggested that VitD and its analogs were potentially useful for MS control. Ensuing publications have revealed mechanistic aspects associated with protection including a reduction in leucocyte infiltration in the CNS, inhibition of Th17 differentiation, and cell apoptosis [29,30,31]. We recently described that an early VitD supplementation beginning 24 h after EAE induction was highly effective in decreasing prevalence, clinical score, inflammation, and demyelination. Of note, VitD administration was able to avoid the characteristic disruption of the BBB permeability that precedes EAE paralysis [32].

A more recent breakthrough for understanding EAE/MS immunopathogenesis was the discovery that the activation of pathogenic Th17 cells in EAE and MS occurs mostly in the small intestine [33,34]. Interestingly, VitD plays a pivotal role in intestinal homeostasis by suppressing Th1/Th2 activation while favoring Treg expansion [35]. Once expanded and activated at the periphery, myelin-specific T cell subsets invade the CNS through the BBB [36,37]. A wave of lymphocyte reactivation occurs locally after the recognition of self-antigens presented by major histocompatibility complex (MHC) molecules at the surface of antigen-presenting cells (APCs) [38]. Once in the CNS, these lymphocytes interact with local (microglia) and infiltrating myeloid cells [monocyte-derived macrophages, neutrophils, and dendritic cells (DCs)], triggering a complex inflammatory process involving inflammasome activation and oxidative stress [39]. The long-term inflammatory process eventually triggers demyelination, axonal damage, and gliosis, leading to progressive aggravation of disability [40,41]. A great deal of attention has been focused on the crucial components that allow transmigration through the BBB in various inflammatory conditions of the CNS and on therapeutic strategies that could block this process [42]. Recently, we demonstrated that VitD and ketotifen were able to reduce BBB disruption significantly controlling the severity of EAE [32,43].

A plethora of drugs have been approved by the US Food and Drug Administration to reduce the number of relapses and mitigate the progression of neurological disability [44]. Even though effective for controlling disease development, these pharmaceuticals are associated with severe side effects such as flushing, chest pain, dyspnea, tachycardia, anxiety, cytokine storm, and increased risk of infections [45]. In this context, other substances are being tested as alternative or adjunct therapies for MS. Due to its antioxidant and immunoregulatory properties [4,46], VitD has been recommended as a prophylactic or therapeutic approach in many inflammatory and autoimmune diseases [47,48]. Much of what is known about the efficacy of VitD in MS derives from experiments with EAE. Most of the literature data support prophylactic or therapeutic effects of VitD on EAE development and this has been attributed to interaction of this hormone with VDR. Protection has been classically associated with its immunomodulatory potential over DCs, macrophages, and T cell subsets [39] but a direct effect on oligodendrocytes differentiation and maturation has also been recently reported [40]. Using the EAE model, we demonstrated that VitD can act as a tolerogenic adjuvant when associated with a peptide from myelin oligodendrocyte glycoprotein (MOG35–55). This ability to induce tolerance was protective in both prophylactic and therapeutic strategies [49,50]. We also tested the tolerogenic potential of paricalcitol (Pari), a vitamin D analog, when administered by an epicutaneous route in association with MOG35–55. This combination successfully controlled EAE severity when applied three and 11 days after EAE induction [51]. We recently observed that an early intervention with VitD, delivered 24 hours after experimental encephalomyelitis induction, prevented neurodegeneration by downmodulating both inflammasome and oxidative stress [32]. Nonetheless, VitD intake at supraphysiological doses can lead to hypercalcemia, and therefore to side effects such as cardiac arrhythmias, renal vasoconstriction, and acute kidney injury [52]. This encouraged the development of many synthetic analogs conceived with similar beneficial properties without the hypercalcemic deleterious effect [53]. One of these non-calcemic analogs is Pari, which has been previously used to treat secondary hyperparathyroidism associated with chronic renal failure [54]. VitD and Pari activities are both triggered by their binding to the vitamin D receptor (VDR) which is present in many cell types including neurons, astrocytes, and immune cells [55]. Evidence is emerging in support of VDR-independent or nonclassical VDR-dependent effects of VitD on immune cells. However, further studies are necessary to address the clinical consequences of these pathways [56].

In this context, the experimental murine model of MS was employed to investigate if VitD and a vitamin D analog, namely paricalcitol, were equally effective as prophylactic therapy when administered at the preclinical disease phase. The most critical stages of EAE development, i.e., the peripheral autoimmune response, the BBB permeability, and the extension of the inflammatory process in the gut and the CNS, were comparatively investigated to reveal possible mechanistic aspects. The effect of these two secosteroidal hormones over the development of DCs derived from murine bone marrow cells was also compared.

## 2. Results

### 2.1. Preclinical Therapy with Vitamin D3 (VitD), but Not with the Vitamin D Analog Paricalcitol (Pari), Controlled Clinical Experimental Autoimmune Encephalomyelitis (EAE) Severity

**Figure 1:**
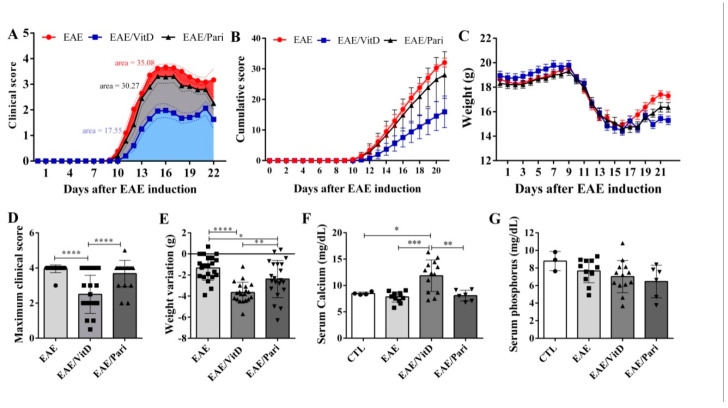
Effect of preclinical administration of vitamin D3 (VitD) and paricalcitol (Pari) on the clinical evolution of experimental autoimmune encephalomyelitis (EAE). C57BL/6 mice subjected to EAE were treated with 8 doses of VitD or Pari every other day by ip route. Treatment begun seven days after encephalomyelitis induction, and clinical score and body weight were daily checked. Blood samples were collected right after euthanasia at 22 days post disease induction. Clinical score (**A**); cumulative score (**B**); daily body weight (**C**); maximum clinical score (**D**); weight variation (**E**); serum levels of calcium (**F**); and phosphorus (**G**). Results are expressed as mean ± SEM, * *p* < 0.05, ** *p* < 0.01, *** *p* < 0.001, **** *p* < 0.0001. (**A**–**E**) represents data from five independent experiments, *n* = 19–22 mice per group. (**F**,**G**) show data from 3 independent experiments for calcium and phosphorus, *n* = 10–13 samples per group (EAE, EAE/VitD and EAE/Pari); except healthy control group (CTL) shows data from 1 independent experiment (*n* = 3–4 samples per group).

As expected, EAE mice developed a severe disease characterized by elevated degree of paralysis (Figure 1A) and accentuated body weight loss (Figure 1C). Treatment with VitD was able to restrain disease evolution. These animals displayed lower clinical (Figure 1A) and cumulative scores (Figure 1B) during the entire experimental period. Instead, Pari therapy was ineffective; these animals developed clinical scores (Figure 1A), cumulative scores (Figure 1B), and maximum clinical scores (Figure 1D) comparable to untreated EAE mice. Marked weight loss was observed in the three experimental groups (Figure 1C), however, this loss was more accentuated in the VitD-treated group (Figure 1E). In addition, only VitD determined an evident hypercalcemia (Figure 1F). No differences were observed in serum phosphorus levels (Figure 1G).

### 2.2. VitD, but Not Pari, Reduced Inflammation and Demyelination in the Central Nervous System (CNS)

**Figure 2:**
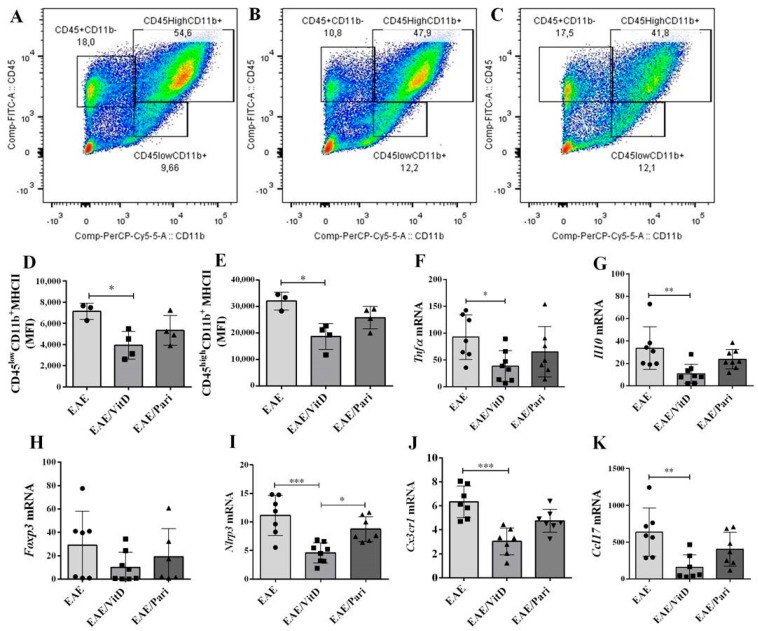
Effect of VitD and Pari on central nervous system (CNS) inflammatory parameters. C57BL/6 mice subjected to EAE were treated with 8 doses of VitD or Pari every other day by ip route. Treatment begun seven days after encephalomyelitis induction and the inflammatory condition of the CNS was determined by cytometry and qPCR on the 22nd day after the induction of the disease. Representative flow cytometry gate strategy for lymphocytes, infiltrating macrophages/activated microglia, and resting microglia [(**A**) = EAE, (**B**) = EAE/VitD, and (**C**) = EAE/Pari] and major histocompatibility complex (MHC) II MFI in infiltrating macrophages/activated microglia and resting microglia ((**D**) and (**E**), respectively). mRNA expression levels of *Tnfa* (**F**), *Il10* (**G**), *Foxp3* (**H**), *Nlrp3* (**I**), *Cx3cr1* (**J**), and *Ccl17* (**K**) were measured at the lumbar spinal cord. Results are expressed as mean ± SEM, * *p* < 0.05, ** *p* < 0.01, and *** *p* < 0.001. (**A**–**E**) data are representative of two independent experiment, *n* = 3 or 4 samples per group. (**F**–**K**) show data from 2 independent experiment (*n* = 6–8 samples per group).

The flow cytometry gate strategy for lymphocytes, infiltrating macrophages/activated microglia, and resting microglia in EAE, EAE/VitD, and EAE/Pari, are depicted in Figure 2A,B,C, respectively. No differences were observed in the percentage of these cell populations among the groups. Clear differences were, however, detected when the degree of activation of infiltrating macrophages and resident microglia was analyzed. Resting microglia (Figure 2D) and macrophages/activated microglia (Figure 2E) eluted from the EAE/VitD group expressed lower MHC II levels than the cells obtained from the two other experimental groups. In addition, the mRNA expression levels of *Tnfa* (Figure 2F), *Il10* (Figure 2G), *Nlrp3* (Figure 2I), *Cx3cr1* (Figure 2J), and *Ccl17* (Figure 2K) were significantly lower in the group treated with VitD. There was no difference in *Foxp3* mRNA expression (Figure 2H).

**Figure 3:**
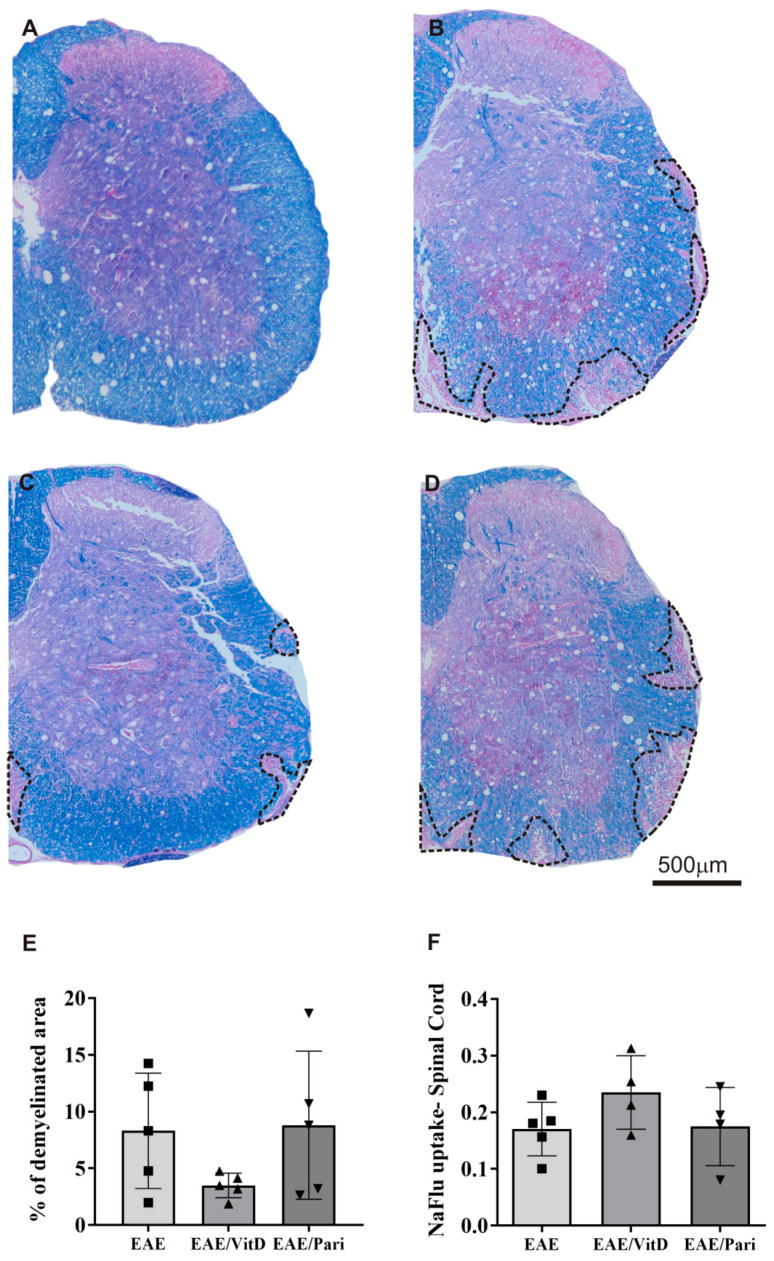
Effect of VitD and Pari on CNS inflammation and demyelination. C57BL/6 mice subjected to EAE were treated with 8 doses of VitD or Pari every other day by ip route. Treatment begun seven days after encephalomyelitis induction and demyelination and BBB permeability were evaluated 22 days after the induction of the disease. Luxol fast blue (LFB) stained sections from normal healthy (**A**), EAE (**B**), EAE/VitD (**C**), and EAE/Pari mice (**D**), quantification of demyelination in the lumbar spine cord LFB-stained sections (**E**), and BBB permeability assay in the spinal cord (**F**). The most clearly demyelinated areas are surrounded by a dotted line. Data are representative of two independent experiment, *n* = 4–5 samples per group, except CTL, *n* = 3 samples.

As expected, no demyelination was detected in healthy control mice (Figure 3A), whereas the lumbar spinal cord anterior and lateral funiculi of the EAE group presented clear demyelinating areas (Figure 3B). Despite treatment, EAE/VitD (Figure 3C) and EAE/Pari (Figure 3D) groups also showed demyelination in the anterior and lateral funiculi. The extension of demyelination found in EAE and EAE/Pari groups was similar, however, the degree of demyelination in the EAE/VitD group showed a tendency to be lower than in the EAE/Pari group, as illustrated in Figure 3E. No difference was observed concerning BBB permeability among the three experimental groups, as represented in Figure 3F.

### 2.3. VitD, but Not Pari, Decreased Cytokine Production by Draining Lymph Nodes Derived Cells

**Figure 4:**
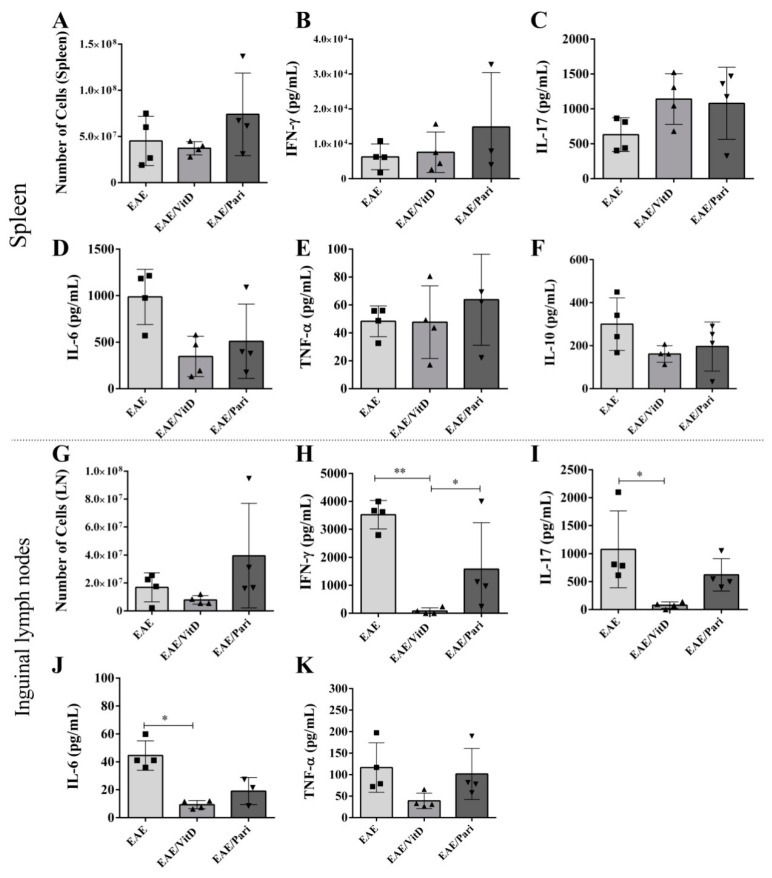
Effect of VitD and Pari treatment on cytokine production by spleen and inguinal lymph node cell cultures. C57BL/6 mice subjected to EAE were treated with 8 doses of VitD or Pari every other day by ip route. Treatment started seven days after encephalomyelitis induction and the production of cytokines by spleen and lymph node cells was determined 22 days after the disease induction. Number of cells (**A**,**G**) and production of IFN-γ (**B**,**H**), IL-17 (**C**,**I**), IL-6 (**D**,**J**), TNF-α (**E**,**K**), and IL-10 (**F**) assessed by enzyme-linked immunoassay in the supernatants of spleen and inguinal lymph node cell cultures stimulated with MOG. Results are expressed as mean ± SEM, * *p* < 0.05, ** *p* < 0.01. Data are representative of two independent experiments, *n* = 4 samples per group.

The cytokine production by the spleen cell cultures was not statistically affected by VitD or Pari, as illustrated for IFN-γ (Figure 4B), IL-17 (Figure 4C), IL-6 (Figure 4D), TNF-α (Figure 4E), and IL-10 (Figure 4F). Instead, the production of IFN-γ, IL-17, and IL-6 by cells from inguinal lymph nodes, as respectively showed in Figure 4H–J, was clearly downmodulated by VitD. These alterations were not found in animals that were treated with Pari. Differences in the number of cells in the spleen and inguinal lymph nodes, among the three experimental groups, were not significant, as shown in Figure 4A,G, respectively.

### 2.4. Gut Inflammation Is Better Controlled by VitD Than by Pari

**Figure 5:**
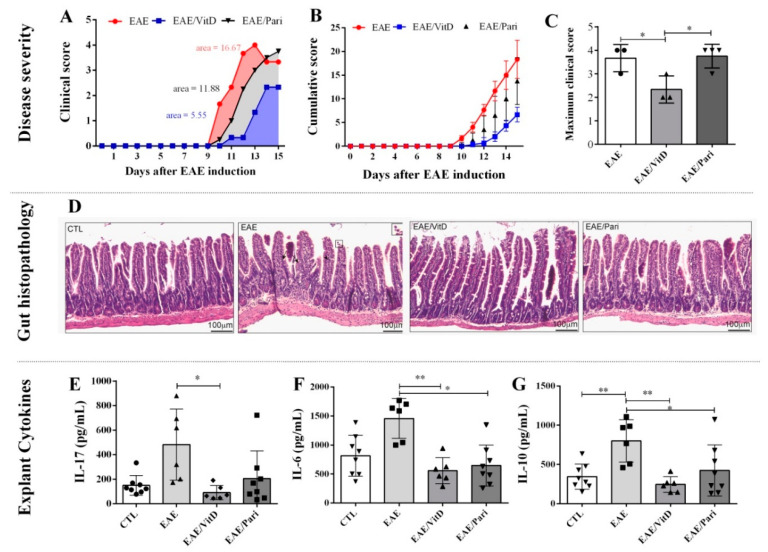
Effect of VitD and Pari on gut inflammation. C57BL/6 mice were treated with VitD or Pari by ip route. Therapy started on the 7th day and ended on the 15th day after EAE induction. Clinical score (**A**), cumulative score (**B**), and maximum clinical score (**C**) were daily evaluated. Histopathological evaluation and cytokine production were analyzed in jejunum and ileum samples collected 15 days after disease induction. Histopathological sections from normal healthy controls, EAE, EAE/VitD and EAE/Pari (**D**). Levels of IL-17 (**E**), IL-6 (**F**), and IL-10 (**G**) produced by gut explants. Results are expressed as mean ± SEM, * *p* < 0.05, ** *p* < 0.01. (**A**–**C**) data are representative of two experiments (*n* = 3–4 samples per group). (**D**) shows data from one experiment (*n* = 4–5 samples per group); (**E**–**G**) show data from one experiment (*n*= 6–8, 2 samples of each animal).

Since proinflammatory T cells home to the gut before they migrate to the CNS during EAE, the animals were euthanized at the 15th day after EAE induction to compare the effect of VitD and Pari in this process. Comparatively to the longer therapy (eight doses), mice treated with four VitD doses were similarly protected from severe paralysis. These animals developed a much less severe disease characterized by reduction in clinical scores (Figure 5A), cumulative clinical scores (Figure 5B), and maximum clinical scores (Figure 5C). The histopathological analysis of sections from the small intestine revealed a mononuclear infiltrate in the lamina propria and submucosa of the EAE untreated mice. These animals also displayed a slight reduction in the villus length along with hyperplasia of the epithelial cells (arrows), and the presence of mononuclear and polymorphonuclear cells in the lumen of the small intestine (upper right insert), which is a hallmark for gut inflammation (Figure 5D). A similar inflammatory process was found in Pari-treated mice but not in CTL and VitD-treated mice, as demonstrated in Figure 5D. Indeed, compared with the EAE mice, the EAE/VitD group displayed a significant improvement in the cell infiltration in the lamina propria, an increase in the villus length, and no recruitment of immune cells to the gut lumen.

As expected, higher levels of IL-17 (Figure 5E), IL-6 (Figure 5F), and IL-10 (Figure 5G) were released by explants from EAE mice compared with CTL animals. IL-6 and IL-10 were downmodulated by both therapies, but these changes were statistically significant in the presence of VitD. Notably, only VitD, but not Pari, significantly reduced IL-17 release (Figure 5D).

### 2.5. VitD and Pari Distinctly Affect the Differentiation of Bone Marrow-Derived Dendritic Cells (DCs)

**Figure 6:**
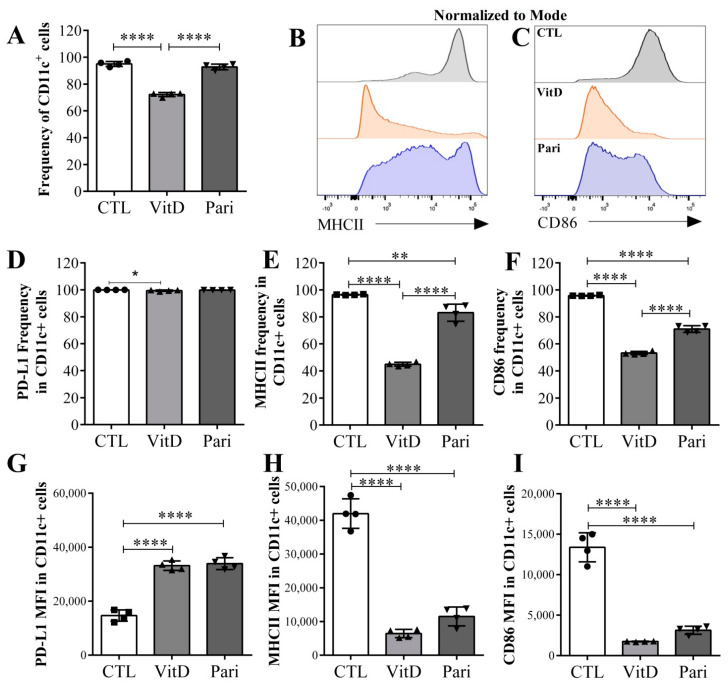
Frequency and phenotype of dendritic cells (DCs) differentiated in the presence of VitD and Pari. DCs were generated from bone marrow precursor cells obtained from C57BL/6 mice. VitD and Pari were added to the culture during the differentiation period. To characterize the DCs, CD11c+ (**A**) were gated based on FSC-A and SSC-A (not shown). Frequency of CD11c+ cells A, histogram of MHC II and CD86 in CD11c+ DCs [(**B**) and (**C**)]. PD-L1 (**D**), MHC II (**E**), and CD86 (**F**) frequencies in CD11c+ DCs. PD-L1 (**G**), MHC II (**H**), and CD86 (**I**) MFI in CD11c+ DCs. Results are expressed as mean ± SEM, * *p* < 0.05, ** *p* < 0.01, and **** *p* < 0.001. Data are representative of two independent experiments, *n* = 4 samples per group.

**Figure 7:**
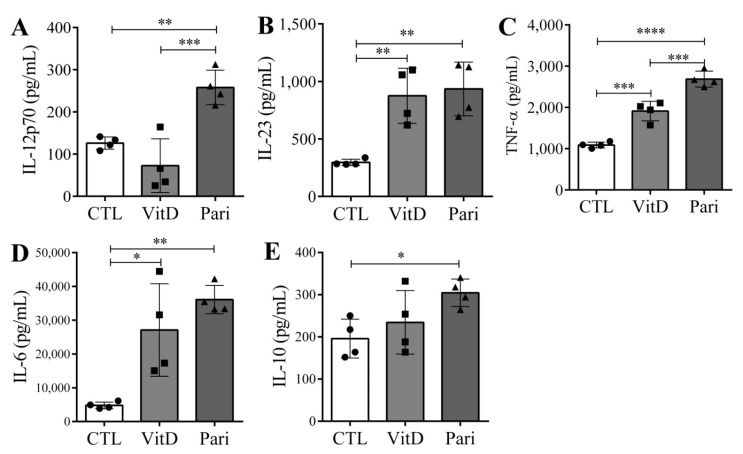
Cytokine production by DCs differentiated in the presence of VitD and Pari. DCs were generated from bone marrow precursor cells obtained from C57BL/6 mice. VitD and Pari were added to the culture during the differentiation period. IL-12p70 (**A**), IL-23 (**B**), TNF-α (**C**), IL-6 (**D**), and IL-10 (**E**) were quantified in DCs supernatant. Results are expressed as mean ± SEM, * *p* < 0.05, ** *p* < 0.01, *** *p* < 0.001, and **** *p* < 0.001. Data are representative of two independent experiments, *n* = 4 samples per group.

Bone marrow cells from C57BL/6 mice were differentiated into DCs in the presence of VitD or Pari. Some phenotypic markers and the production of Th1 and Th17 polarizing cytokines were analyzed after the differentiation process. On the one hand, the frequency of CD11c+ cells differentiated in the presence of Pari was comparable to that found in the CTL condition, in the absence of Pari or VitD. On the other hand, the addition of VitD to the cultures reduced the frequency of fully differentiated DCs (Figure 6A). The percentage of CD11c+ cells expressing MHC II (Figure 6B,E) and CD86 (Figure 6C,F) was reduced by both, VitD and Pari, however, downregulation determined by VitD was significantly more accentuated than by Pari. Mean fluorescence intensity (MFI) of MHC II and CD86 was similarly reduced by VitD and Pari, as displayed in Figure 6H,I, respectively. Even though all CD11c+ cells expressed PD-L1 (Figure 6D), VitD and Pari equivalently increased PD-L1 expression (Figure 6G). VitD and Pari presence also contrastingly affected cytokine production. Pari addition to the cultures significantly increased all tested cytokines, i.e., IL-12p70, IL-23, TNF-α, IL-6, and IL-10, as shown in Figure 7A,B,C,D,E, respectively. Instead, the presence of VitD only increased the production of IL-23, TNF-α, and IL-6, and the production of IL-12p70 and IL-10 was not affected.

## 3. Discussion

The possible use of VitD as an add-on therapy to the previously well-established MS disease-modifying therapies was initially based upon epidemiological data indicating lower levels of this vitamin in patients and from its immunomodulatory potential [4,17]. Evidence that VitD reaches the CNS, protects neurons against both neuroinflammation and oxidative stress [57], and stimulates remyelination [58] reinforces its therapeutic potential. In this scenario, many clinical trials have been previously performed. Nonetheless, there is still a consistent controversy about its clinical application in MS [59,60]. Our own experience with VitD in EAE indicates that it is very effective when administered near the disease induction period [32,49,50]. In this context, we asked if VitD would still be effective if administered after the disease induction phase when the priming of myelin-epitope-specific CD4+ T cells has already been established. VitD effect was also compared with Pari because it is endowed with immunomodulatory activities and is also one of the low-calcemic vitamin D analogs.

Initially, we observed that VitD, but not Pari, was able to significantly control the clinical disease severity. This contrasting clinical efficacy between VitD and Pari was also confirmed by a direct CNS analysis. The percentage of lymphocytes, macrophages/activated microglia, and resting microglia was comparable among the three experimental groups. However, local inflammation was clearly decreased in the VitD-treated animals. The level of MHC II and CD86 expression on the surface of infiltrating macrophages and microglial cells, for example, was clearly downregulated by VitD but not by Pari. This seems quite relevant because the contribution of MHC II and CD86 to EAE and MS immunopathogenesis, by locally processing and presenting self-antigens, is well established [61]. In addition, the therapeutic effect of other candidate molecules for MS therapy, such as minocycline [62] and berbamine [63], was associated with reduced expression of these molecules in the CNS.

Other findings in the CNS such as significantly lower mRNA levels encoding *Tnfa*, *Nlrp3*, *Cx3cr1*, and *Ccl17* confirmed the stronger VitD downmodulatory ability when compared with Pari. Several lines of evidence support the contribution of these molecules to EAE/MS immunopathogenesis and also their modulation by VitD. The role of TNF-α in MS/EAE pathogenesis is complex and includes the following two opposite aspects: essential maintenance of the local immune homeostasis and a detrimental effect in the CNS environment [64]. Despite this, amelioration of EAE severity by administration of alternative therapies, including VitD, has been associated with decreased production of this cytokine [65,66]. Reduction in the activation of the inflammasome platform, indicated by diminished expression of *Nlrp3*, was also coherent with the pivotal role played by this system in the MS/EAE inflammatory process. Evidence in patients with MS suggests that inflammasome activation occurs during disease. Experiments with EAE specifically point to NLRP3 inflammasome protein as critical and necessary for disease development [39]. Reduced activation of this system in EAE by therapy with ghrelin has been reported [67]. The significantly impaired expression of *Cx3cr1* by VitD is worth mentioning because one of the main roles of this receptor is to allow leukocyte’s recruitment to the CNS [68]. The relevance of this function is illustrated by the demonstration that pharmacological inhibitors of CX3CR1 attenuated disease severity in EAE [69]. According to the literature, this decreased expression of *Cx3cr1* could be involved in the mechanism by which VitD played a protective effect or, alternatively, could be a consequence of the reduced inflammatory process. The promoting role of the chemokine CCL17 in EAE has been partially attributed to its ability to allow the immigration of IL-17-producing CD4+ T cells and peripheral DCs to the CNS [70]. We have previously observed this reduced mRNA expression of *Nlrp3* and *Ccl17* in the CNS of mice treated with VitD right after EAE induction [32].

The decrease in the local inflammatory parameters coincided with a clear tendency towards less demyelination in the VitD-treated mice. This was considered meaningful because damage to the myelin sheath will lead to irreversible axonal damage and loss, and therefore to physical disability [71]. We previously demonstrated that supplementation with VitD initiated very early, 24 h after EAE induction, was very effective and able to preserve BBB permeability [32]. In this investigative study, the BBB permeability was also checked to clarify if this delayed VitD supplementation that was still able to promote significant protection was concomitant to BBB preservation. However, in this case, VitD did not preserve BBB permeability, therefore, ruling out the contribution of this mechanism of protection.

Even though VitD therapy started seven days after EAE induction, it still downmodulated cytokine production by draining lymph nodes but not by spleen cells. Considering that cell cultures were stimulated with MOG, most of these cytokines were probably produced by autoreactive T lymphocytes. Even though differences in cytokine production between lymph nodes and spleen in EAE have been previously described [72] and as the number of cells in the regional lymph nodes was also significantly reduced in these animals, it is possible that these cells had migrated to other lymphoid organs or to the lungs before they reached the CNS. This hypothesis is based on the phenomenon, namely licensing, according to which T lymphocytes temporarily reside in the lungs where they reprogram their gene-expression profile to be able to express migratory properties that will allow them to reach the CNS [73]. According to some authors this licensing process could occur in other lymphoid organs such as the spleen, intestine, and leptomeninges [37,74,75].

A major breakthrough in EAE/MS was the realization that there is a straightforward correlation among higher frequency of intestinal Th17 cells, dysbiosis, and worse disease activity [34]. The evidence that blocking the entry of T cells into the gut would confer protection against adoptive EAE was also relevant [76]. In this scenario, and considering the critical effects of VitD on the differentiation and bioactivity of Th17 cells [77], we evaluated whether this differential efficacy between VitD and Pari involved differences in gut inflammation and IL-17 production. As Th17 differentiation in the gut precedes the acute clinical disease phase, this was assessed after four doses of both drugs. Even though histopathological evaluation did not show striking differences in inflammation, once again VitD, but not Pari, significantly decreased IL-17 production.

Finally, to further understand the differences between VitD and Pari, we compared in vitro effects of VitD and Pari on DC differentiation, focusing on phenotypic markers and cytokine production. Considering both parameters, we believe that VitD presence during DC differentiation polarized these cells to a tolerogenic phenotype. This did not happen in the presence of Pari. The tolerogenic potential of VitD through DC modulation is widely supported by the literature [78,79]. Otherwise, knowledge regarding the effect of Pari on DC differentiation is limited. The two available publications [80,81] indicate, in contrast to our findings, that Pari generates tolerogenic DCs. A noteworthy difference between their procedure and ours was the origin of DCs; they differentiated DCs from human monocytes, whereas we used murine bone marrow precursors. Therefore, it is possible that Pari activity over DCs is less pronounced in murine than in humans.

Altogether our findings indicated that Pari, differently from VitD, was not effective as a preclinical prescription to reduce EAE severity. This failure was unexpected considering that previous publications attested protective ability by other vitamin D analogs [82,83]. In addition, we have previously demonstrated that Pari efficiently controlled EAE severity when applied by epicutaneous route (patch procedure) as a tolerogenic adjuvant, that is, associated with the autoantigen-MOG [51]. This failure could be linked to the fact that therapy was initiated only at the preclinical disease phase, at Day 7 after disease induction, when the autoimmune response was already well established. In this case, even VitD itself was less effective as compared with a more precocious therapy start, i.e., 24 h after EAE induction [32]. A very recent publication by Zhang et al. (2020) [84] gives some support to this interpretation considering that they started therapy with Pari 48 h after disease induction. This possible correlation between disease evolution time and Pari efficacy is also reinforced by the finding that mice treated with this analog before being induced to acute-depression-like behaviors by systemic LPS injection, developed a much less severe disease, possibly by repressing local NF-kB activity and NLRP3 activation. The ability of a vitamin D analog to control EAE severity was demonstrated by Garcion et al. (2003) [85] by using a Lewis rat model of chronic relapsing disease treated with MC1288. The fact that Pari is an analog of vitamin D2 and not VitD could also be relevant. It has been described that calcitriol and Pari trigger distinct profiles of gene transcription after its interaction with VDR [86]. The differential levels of IL-6 and IL-12p70 triggered by the addition of these two substances illustrates this possibility.

Translation of data from preclinical models to humans is a troublesome task due to the intrinsic characteristics of each species. However, nowadays, it is still accepted as necessary, in view of the ethical issues to test new procedures directly in human subjects. Even though most preclinical assays indicated a strong potential of VitD as useful for EAE prevention or therapy, clinical trials with patients revealed mixed data [87]. Despite the complexity of this subject, we believe that more clinical assays are worthwhile to be conducted and could clarify this controversy. In this sense, and based on that and another investigation by our team [32], we postulate that the sooner the supplementation the better the effect. Considering our experience, a very precocious prophylactic therapy initiated one day, but not seven days after EAE induction, was able to control most of BBB disruption which is a critical event for disease development. We also believe that preclinical assays would be more relevant if carried out by oral supplementation that directly mimics the friendliest route of administration in humans. The fact that VitD causes hypercalcemia cannot be neglected because of its pathophysiological consequences. In this sense, in this investigation, we showed that the vitamin D structural analog Pari, administered by the same protocol as VitD, displayed less pronounced immunomodulatory ability and was not capable of controlling EAE development. This finding points to the need to thoroughly investigate and compare the numerous available vitamin D analogs concerning their immunomodulatory potential. In this sense, gene expression profiling directly with freshly isolated human peripheral blood mononuclear cells could be enlightening. The relevance of VitD levels and its potential use as an adjunct therapy in other demyelinating diseases that also affect the CNS, for example, neuromyelitis optica and acute disseminated encephalomyelitis, is still a fully open area for investigation [88,89].

In summary, our data highly suggest that there is a window of opportunity for VitD treatment in EAE that seems to be even more critical when the therapy is done with the analog Pari. Our results indicate that VitD efficacy still takes place if therapy is postponed to the preclinical disease stage, even though its effect is less stringent as compared with more precocious or even prophylactic measures as we and other authors have previously demonstrated. Distinct mechanisms are probably contributing to this therapeutical effect including modulation of peripheral and possibly local immune response, direct anti-inflammatory effects at the CNS, and decreased Th17 differentiation in the gut. Regarding Pari, our findings suggest that it is not a suitable substitute for treatment with VitD in this disease, at least in a comparable VitD administration regimen. However, we do not exclude the possibility that this VitD analog could avoid EAE development if administered before or soon after disease induction.

## 4. Materials and Methods

### 4.1. Experimental Design

**Figure 8:**
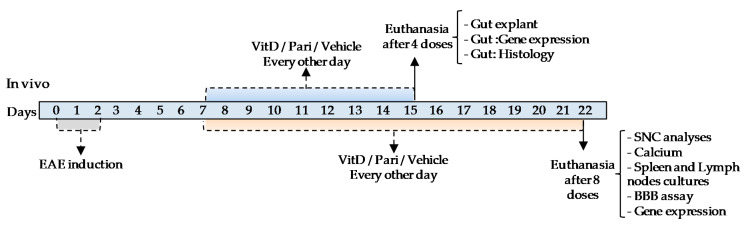
In vivo experimental design timeline.

Most of the analyses were done with samples obtained from mice submitted to EAE induction. In this case, the animals were allocated into the following four groups: CTL; EAE (submitted to disease induction and injected with the vehicle); EAE/VitD; and EAE/Pari (submitted to disease induction and treated with VitD or Pari, respectively). The following therapeutic schedule was elected for most assays: 8 ip doses of VitD, Pari or vehicle, every other day beginning 7 days after EAE induction. Weight and clinical score were evaluated daily and most of the other analyses were performed 24 h after the last therapeutic dose, 22 days after EAE induction. Prior to biological samples collection, the animals were anesthetized with ketamine/xylazine, the blood was obtained by cardiac puncture, and the animals were transcardiacally perfused with 10 mL of cold PBS. Serum samples were employed for calcemia and phosphatemia quantification, inguinal lymph nodes and spleen cell cultures for cytokine quantification, and CNS for histopathological analysis and expression of MHC II levels and inflammatory parameters. These analyses were done after 8 therapeutic doses of VitD or Pari, every other day, based mainly on the classical publication of Lemire and Archer, 1991 [27] and on our last investigation [32]. This schedule allowed a direct comparison with an earlier therapy initiation which proved to be highly effective. The assays concerning cytokine release by the gut and its histopathological analysis were done with samples obtained after 4 doses of VitD and Pari, at the 15th day after EAE induction. We opted for a more precocious analysis, after 4 doses as the literature revealed that gut involvement precedes paralysis manifestations [76]. A timeline of this in vivo schedule is displayed below (Figure 8).

**Figure 9:**
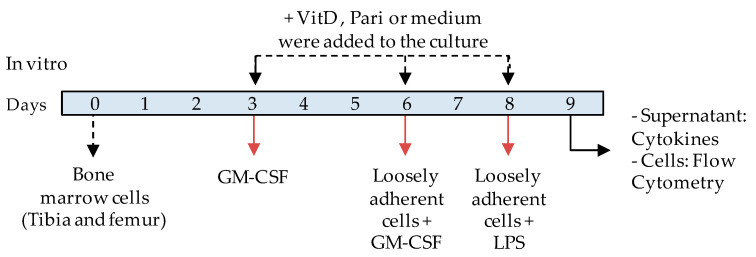
In vitro experimental design timeline.

The effect of VitD and Pari on DC differentiation was analyzed in vitro by using cell cultures with precursors obtained from C57BL/6 bone marrow. A timeline scheme indicating the addition of the reagents to the cultures is displayed below (Figure 9).

### 4.2. Animals

C57BL/6 female mice, 6 weeks old, were purchased from the University of São Paulo (USP, Ribeirão Preto, SP, Brazil) and acclimated to UNESP animal facility for 2 weeks before disease induction. The animals received sterilized food and filtered water ad libitum. The experiments were carried out according to the regulations of the Ethics Committee on Animal Use, CEUA, Institute of Biosciences, Botucatu (protocol number #1099).

### 4.3. Induction and Clinical Assessment of EAE

C57BL/6 mice were injected subcutaneously, in the back, with 100 µg of MOG_35–55_ (Genemed Synthesis Inc., San Antonio, TX, USA) emulsified in 50 µL of CFA (Millipore-Sigma, St Louis, MO, USA) containing 4 mg/mL heat killed of *Mycobacterium tuberculosis.* On the day of disease induction and 48 h later, the animals were injected with 200 ng of pertussis toxin (Millipore-Sigma) by ip route. Weight and clinical disease signs were daily evaluated. The severity of the disease was defined according to the following scale: no symptoms (0), limp tail (1), hind leg weakness (2), partially paralyzed hind legs (3), complete hind leg paralysis (4), and complete paralysis (5). Body weight variation was calculated by the difference between the weight at the euthanasia day and the weight determined just before disease induction.

### 4.4. Therapy with VitD and Pari

Mice were treated with 8 or 4 doses of 0.1 ug/day of vitamin D3 (D1530- Millipore-Sigma) or paricalcitol (Zemplar^®^, Abbott Laboratories, Chicago, IL, EUA) by ip route every other day, starting 7 days after EAE induction. The CTL and EAE group were injected with the vehicle in the same frequency.

### 4.5. Serum Calcium and Phosphorus

Blood samples collected after anesthesia were centrifuged, and the sera stored at −20 °C until further analyses. Serum levels of calcium and phosphorus were measured according to the instructions of the manufacturer (Cálcio Arsenazo III and Fósforo UV, Bioclin-Quibasa Química Básica Ltda, Belo Horizonte, MG, Brazil).

### 4.6. Spleen and Lymph Node Cell Cultures

Inguinal lymph nodes were removed, disaggregated on cell strainers, and the cells resuspended in complete RPMI 1640 medium (10% heat-inactivated fetal bovine serum (FBS, Gibco-Thermo Fisher Scientific, Waltham, MA USA), 1% of gentamicin (Millipore-Sigma), and 4 mM of L-glutamine (Millipore-Sigma). Spleens were removed and disaggregated on cell strainers, the erythrocytes were lysed with an NH_4_Cl solution (0.15 M) and the cells were resuspended in complete RPMI medium. Spleen and lymph nodes cells were adjusted to 5 × 10^6^ cells/mL and 2.5 × 10^6^ cells/mL, respectively. Both cultures were re-stimulated in vitro with MOG35–55 (20 mg/mL) and incubated at 5% CO_2_/37 °C for 48 h. then, culture supernatants were collected and stored at −20 °C for further cytokine quantification.

### 4.7. CNS Mononuclear Cell Isolation

Brain and the entire spinal cord were collected and digested with 2.5 mg/mL of collagenase D (Roche Applied Science, Indianapolis, IN, USA) and 100 µg/mL of DNAse (Millipore-Sigma) at 37 °C for 45 min. After maceration, the homogenates were washed in Hank’s Balanced Salt Solution (HBSS) (Gibco-Thermo Fisher Scientific), resuspended in 30% Percoll (GE Healthcare, Uppsala, Sweden), and gently laid over 70% Percoll. After centrifugation at 950× *g* for 20 min with the centrifuge brake off, the two gradient Percoll interface containing the mononuclear cells was collected and washed twice (450× *g*/7 min) with HBSS. The cells were adjusted to 10^6^ cells/mL to perform the flow cytometry analyses.

### 4.8. Blood–Brain Barrier Permeability Assay

The BBB permeability was performed as previously described by Christy et al. (2013) [90]. Mice were injected by ip route with 10% sodium fluorescein solution (NaFlu, Millipore-Sigma). After 20 min, they were anesthetized to withdraw blood samples through heart puncture, and then perfused with 10 mL of 0.9% saline solution. The whole spinal cords were collected, weighed, macerated with pestles in the presence of 400 mL of saline solution, and then centrifuged (8600 *g*, 22 °C for 10 min). A 100 uL of supernatants and plasma samples were distributed in a 96-well black plate and read in a Synergy 4 fluorimeter (BioTek Instruments, Winooski, VT, USA). NaFlu uptake by the spinal cord was calculated according to the following equation: (tissue sample RFU/tissue sample weight)/(plasma RFU/amount of cardiac blood). RFU, relative units of fluorescence.

### 4.9. Spinal Cord and Gut Histopathology

Lumbar spinal cord and gut samples (jejunum and ileum) were fixed in 10% neutral buffered formalin, dehydrated in graded ethanol, and then included in Paraplast Plus (McCormick, St. Louis, MO, USA). Five μm thick sections from the spinal cord and gut samples were obtained using a Leica RM2245 microtome. Then, spinal cords were stained with LFB to quantify the extent of demyelination. Gut samples were stained with hematoxylin and eosin to evaluate the inflammation. Demyelination was evaluated in a bright-field microscope (Axioplan2, Carl Zeiss Microscopy GmbH, Oberkochen, Germany) in a 10× objective lens (NA = 0.25) attached to a digital camera (AxioCamHRc, Carl Zeiss). Sections were photographed and analyzed in the ImageJ software [Schneider, Rasband et al. (2012)] (version 1.49, U.S. National Institutes of Health). Total cross-section area of white matter and demyelination areas were measured, and demyelination percentage was calculated as follows: (demyelination area/white matter area) × 100.

### 4.10. Gut Explant-Immersion Cultures

This procedure was based on two previous reports [91,92]. The entire jejunum and ileum were aseptically removed and cleaned by infusing cold HBSS containing antibiotic/antimycotic (1% Millipore-Sigma). Then, the cleaned sections (1.0 cm) were incised longitudinally and positioned with the serosa facing down in a 24-well culture plate, with two sections per well. Then, 0.5 mL RPMI 1640 medium containing 10% FBS and 1% antibiotic/antimycotic were added to each well. Next, these cultures were incubated at 37 °C and 5% CO_2_ for 6 h. Then, culture supernatants were collected and stored at −20 °C for further cytokine quantification.

### 4.11. In Vitro Generation of Bone Marrow-Derived DCs

Generation of bone marrow-derived DCs was conducted, as previously described by [42]. Briefly, bone marrow cells were collected from femurs and tibias of 8- to 12-week-old C57BL/6 naïve mice, with the influx of RPMI 1640 medium The 2 × 10^6^ cells were plated in 90 × 15 mm Petri dishes containing 10 mL of RPMI medium supplemented with 20 mg/L of gentamicin, 2% of L-glutamine, 10% FBS, and 50 µM of 2-β-mercaptoethanol (Amersham BioSciences, Buckinghamshire, UK), and then incubated in a 5% CO_2_ incubator at 37 °C. On Day 3, medium was removed and replaced by fresh RPMI supplemented medium and 20 ng/mL of recombinant mouse GM-CSF (Peprotech, Rocky Hill, NJ, USA). On Day 6, loosely adherent cells were collected, centrifuged (400× *g*, 4 °C, 5 min), and incubated in new Petri dishes containing fresh supplemented RPMI medium and rmGM-CSF. On Day 8, loosely adherent cells were collected and activated in fresh supplemented medium containing 10 ng/mL of LPS (O111:B4, Millipore-Sigma) and incubated. The effect of VitD or Pari presence was investigated by their addition to the cultures on Days 3, 6, and 8. On Day 9, 24 h after LPS activation, loosely adherent cells were collected and centrifuged; supernatants were stored at −20 °C for subsequent cytokine analysis, and cells were resuspended in supplemented medium and prepared for flow cytometry analysis.

### 4.12. Flow Cytometry

Flow cytometry staining was performed according to eBioscience-Thermo Fisher Scientific (Waltham, MA, USA) instructions. Samples were acquired on the FACSCanto II flow cytometer (Becton Dickinson, San Jose, CA, USA) from the Department of Chemical and Biological Sciences, Institute of Biosciences, UNESP, Botucatu, Brazil. Cell population characterization was performed by FlowJo software (Version 10, Tree Star, Ashland, OR, USA). CNS cell immunostaining was done with the following labeled antibodies: anti-CD11b-PerCP-Cy5.5 (M1/70), anti-CD45-FITC (30-F11), anti-MHC II-APC (MS/114.15.2), anti-CD40-PE (1C10). Bone marrow-derived DCs were stained with the following antibodies: anti-CD11c-FITC (N418), anti-MHC II-APC (MS/114.15.2), anti-CD86-PE (GL1), and anti-PD-L1-PE-Cy7 (MIH5).

### 4.13. Cytokine Quantification

Cytokine quantification in cell culture supernatants was performed by enzyme-linked immunosorbent assay (ELISA), according to the manufacturer’s instruction. The following cytokines were evaluated: IL-17, IL-6, TNF-α, IL-12p70, and IL-23 (DuoSets R&D Systems, Minneapolis, MN, USA) and IL-10 and IFN-γ (OptEIA sets, BD Biosciences, San Jose, CA, USA).

### 4.14. Rt-PCR and qPCR

Total RNA was extracted from the lumbar spinal cord samples with TRIzol™ reagent (Life Technologies, Austin, TX, USA). The cDNA synthesis was performed using the High-Capacity cDNA Reverse Transcription Kit (Applied Biosystems, Foster City, CA, USA). Then, 12.5 ng of cDNA were submitted to real-time PCR reaction using TaqMan™ Gene Expression Assay (Life Technologies), according to the manufacturer’s instructions. The expression of the following genes was evaluated: *Ccl17*, *Foxp3*, *Cx3cr1*, *Nlrp3*, *Tnfa*, and *Il10*. The mRNA relative expression was determined using the equation 2^-ΔΔCt^. This equation is based on the difference in the cycle threshold (ΔCt) for target and reference genes, divided by the Ct for the calibrator (2^-ΔΔCt^), considering the mean of CTL as the calibrator.

### 4.15. Statistical Analysis

In the case of parametric variables, the comparison among the groups was performed by ANOVA followed by Tukey post hoc test. For nonparametric variables, the comparison was performed by the Kruskal–Wallis test followed by the Dunn’s test. The area under the curve method was adopted to calculate the area in the clinical score graph. Data was presented in mean ± standard deviation and the level of significance was 5%. Data was analyzed by the statistical package GraphPad Prism 6.0 (Version 6.0, GraphPad Software, San Diego, CA, USA).

## Abbreviations

APC	Antigen presenting cells
BBB	Blood–brain barrier
CTL	Healthy control group
CNS	Central nervous system
CFA	Complete Freund’s adjuvant
DC	Dendritic cell
ELISA	Enzyme-linked immunosorbent assay
EAE	Experimental autoimmune encephalomyelitis
LFB	Luxol fast blue
MS	Multiple sclerosis
MOG	Myelin oligodendrocyte glycoprotein
MHC	Major histocompatibility complex
Pari	Paricalcitol, 19-nor-1,25-dihyroxyvitamin D2
VDR	Vitamin D receptor
VitD	Vitamin D3, 1,25-dihydroxyvitamin D3, calcitriol
